# A comprehensive review of training methods for physical demands in adolescent tennis players: a systematic review

**DOI:** 10.3389/fphys.2024.1449149

**Published:** 2024-09-13

**Authors:** Yuxin Guo, Jia Xie, Gengxin Dong, Dapeng Bao

**Affiliations:** ^1^ China Institute of Sport and Health Science, Beijing Sport University, Beijing, China; ^2^ Research Department of Physical Education, Xinjiang Institute of Engineering, Urumqi, China; ^3^ School of Sport Medicine and Physical Therapy, Beijing Sport University, Beijing, China

**Keywords:** physical demands, adolescent tennis, neuromuscular training, functional training, traditional strength training, high-intensity interval training, plyometric training

## Abstract

**Background:**

Adolescent tennis players encounter critical physical demands, but the lack of comprehensive analysis of training types hampers the selection of optimal training programs. This study aims to conduct a systematic literature review to analyze the effectiveness and limitations of various training types on the physical demands of adolescent tennis players, summarizing the optimal training methods to enhance these physical qualities.

**Methods:**

From March 2024, a comprehensive search was conducted across four electronic databases: SCOPUS, PubMed, EBSCOhost (SPORTDiscus), and Web of Science. Additionally, Google Scholar and other sources of gray literature were referenced. Original research articles with an experimental design were included. The methodological quality of the included studies was evaluated using the Physiotherapy Evidence Database (PEDro) scale, and the overall scientific evidence was determined through the best evidence synthesis (BES).

**Results:**

Eighteen articles on exercise training met all inclusion criteria and were included in this systematic review. These studies maintained a high standard of quality, making their findings relatively credible. Among them, five studies investigated plyometric training, five focused on neuromuscular training, three explored functional training, two examined traditional strength training, and three assessed High-Intensity Interval Training.

**Conclusion:**

To enhance speed, strength, power, agility, and dynamic balance, it is recommended to prioritize plyometric training, neuromuscular training, and functional training over traditional tennis training. Functional training is particularly effective for improving flexibility and balance, while plyometric training is more suited for increasing power and speed. Neuromuscular training, when performed before routine workouts, is beneficial for enhancing speed, flexibility, and strength. Hard surface training is ideal for boosting power, whereas sand training excels in improving strength, speed, and balance. Combining HIIT with strength training is especially advantageous for enhancing short-distance sprinting, repeated sprint ability, and power. By appropriately combining and utilizing these training methods, the physical capabilities and sports performance of adolescent tennis players can be comprehensively optimized.

**Systematic Review Registration:**

https://www.crd.york.ac.uk/prospero/, identifier CRD42024578147.

## Introduction

Tennis, recognized as the second-most popular global sport after soccer (Pluim et al., 2023), is a high-intensity intermittent sport that demands high levels of technical, tactical, psychological, and physical demands. Over the past decades, the sport has seen increased professionalization and commercialization, attracting more participants to tennis training (Brouwers et al., 2015). The sport’s rigorous requirements for stroke performance make systematic training and development essential.

For adolescent tennis players, technical skills and physical demands are critical to securing victories. Post-age 12, as players experience increases in height and muscle mass, these physical demands become pivotal in match outcomes (Barbaros et al., 2023). Tennis necessitates exceptional speed, strength, power, agility, and dynamic balance, essential for proficient serving and hitting (Lambrich and Muehlbauer, 2022). Adolescent tennis players can reach a maximum sprint speed of 17.4 km/h during matches, requiring over 100 accelerations per match, with an average of only 4.9 rallies per match and a rally duration of 8.3 s (Pluim et al., 2023). Superior speed enables adolescent tennis players to quickly position themselves during fast-paced matches, while strength helps stabilize their bodies for precise and powerful returns. Formidable power enhances the penetrating force of serves and baseline shots, providing a significant competitive edge. Advanced agility enhances footwork control, allowing players to swiftly adjust their posture and maintain balance during volleys and direction changes. Dynamic balance helps players maintain body control, ensuring stability even under pressure (Pluim et al., 2023; Caballero et al., 2021; Turner et al., 2023). Adolescence is a sensitive phase for developing these physical demands, and strategically combined training during this period can optimize the cyclical advancement of athletic demands (Lloyd and Oliver, 2012).

Current training methods for adolescents in tennis include Plyometric, Neuromuscular, Functional, Traditional Strength, and High-Intensity Interval Training (HIIT). Research shows that plyometrics and combined training enhance neural and muscular functions, improving physical demands (Deng et al., 2024). Neuromuscular training, involving exercises with regular short rests, is effective for boosting athletic demands (Lin et al., 2022). Functional training enhances sport-specific strength, flexibility, balance, and coordination (Yildiz et al., 2019). Resistance training increases maximal strength and force demands (Guo et al., 2022). While HIIT improves overall physical demands and metabolism, its effects on the neuromuscular system are less pronounced (Stankovic et al., 2023). Despite these insights, there is a lack of comprehensive summary and evaluation of various training approaches, presenting a challenge for coaches and athletes in selecting optimal training programs.

Considering the current gap in comprehensive analyses of physical training methods for adolescent tennis players, this study proposes a systematic review to thoroughly examine the effects of various exercise regimes on their physical demands. Through a detailed analysis of current literature, we aim to clarify the efficacy and constraints of each training method. This comprehensive understanding will serve as a scientific guide for coaches and athletes, and set a baseline for future investigations in enhancing training strategies for young tennis players.

## Materials and methods

### Protocol

The protocol for this systematic review adhered to the guidelines set forth by the Preferred Reporting Items for Systematic Reviews and Meta-Analyses (PRISMA) and was registered in PROSPERO with the registration number CRD42024578147.

### Search strategy

A comprehensive literature search was conducted using SCOPUS, PubMed, EBSCOhost (SPORTDiscus), and Web of Science. The search was conducted for all articles related to the physical demands of adolescent tennis players up to 31 March 2024. A Boolean search syntax using the operators” AND” and “OR” was applied. The keywords “training*”, “exercise*”, “intervention*”, ”physical training*”, “exercise training*”, “resistance training*”, “strength training*”, “aerobic exercise*”, ”power training*”, “fitness training*”, “endurance training*”, “conditioning training*”, “physical therapy*”, “adolescent*”, ”teen*”, “youngsters*”, “junior*”, “teenager*”, ”boy*”, ”girl*”, ”tennis*” were utilized. An example of a PubMed search is as follows {[“training*” (Title/Abstract) OR “exercise*” (Title/Abstract) OR “intervention*” (Title/Abstract) OR ”physical training*” (Title/Abstract) OR “exercise training*” (Title/Abstract) OR “resistance training*” (Title/Abstract) OR “strength training*” (Title/Abstract) OR “aerobic exercise*” (Title/Abstract) OR ”power training*” (Title/Abstract) OR “fitness training*” (Title/Abstract) OR “endurance training*” (Title/Abstract) OR “conditioning training*” (Title/Abstract) OR “physical therapy*” (Title/Abstract)] AND [“adolescent*” (Title/Abstract) OR ”teen*” (Title/Abstract) OR “youngsters*” (Title/Abstract) OR “junior*” (Title/Abstract) OR “teenager*” (Title/Abstract) OR ”boy*” (Title/Abstract) OR ”girl*” (Title/Abstract)] }AND [”tennis*” (Title/Abstract)]. Additionally, a manual search was conducted on Google Scholar and through reference lists to ensure no pertinent articles were overlooked. The data-gathering process was supported by skilled librarians to ensure accuracy and completeness.

### Eligibility criteria

Only scholarly literature published in English was included in this review, regardless of the year of publication. Studies had to meet the criteria outlined in the PLCOS framework to be considered for analysis. Table 1 presents the inclusion and exclusion criteria for the systematic review. Studies were included if they met the following criteria (Pluim et al., 2023): the study must be a peer-reviewed publication in English, discussing a randomized controlled trial (RCT) or a non-RCT assessing the effect of exercise training interventions on physical demands (Brouwers et al., 2015); participants must be young tennis players (both male and female) (Barbaros et al., 2023); the study must have assessed at least one component of physical performance, such as speed, strength, power, agility, or dynamic balance (Lambrich and Muehlbauer, 2022). The study must include an intervention group that utilized a combination of one or more training methods, such as Plyometric, Neuromuscular, Functional, Traditional Strength, or HIIT.

**TABLE 1:** Inclusion criteria based on PICOS (population, intervention, comparison, outcome and study design). RCT, randomized controlled trial.

PICOS detail	
Population	Young tennis player (male/female, age 8–18 years old)
Intervention	Exercise training
Comparison	Multiple and single-group trials
Outcome	Physical demands components (speed, power, strength, endurance, flexibility, agility)
Study designs	RCT or Non-RCT

Studies were excluded if they met any of the following criteria (Pluim et al., 2023): included young athletes from sports other than tennis (Brouwers et al., 2015); combined exercise training interventions with other non-exercise training methods or included unsupervised training sessions (Barbaros et al., 2023); were observational studies or interventions focused solely on providing counseling for exercise training implementation; and (Lambrich and Muehlbauer, 2022) were published in languages other than English, including conference abstracts, letters to the editor, case reports, and brief communications.

### Study selection

**FIGURE 1:**
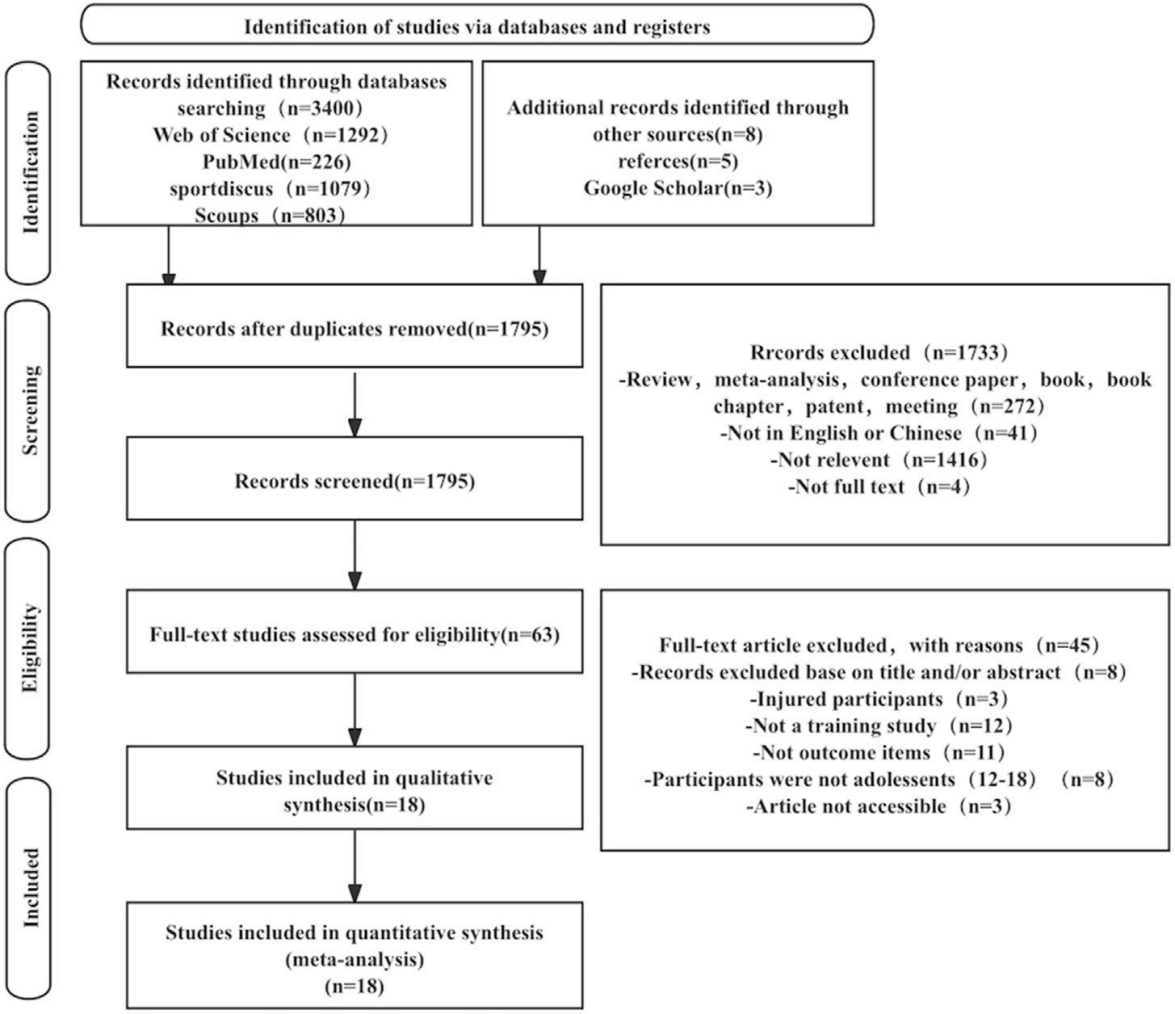
Study selection flow diagram.

After searching four international electronic databases, the information about the retrieved studies (i.e., title, author, and year) was uploaded into Zotero reference management software to eliminate duplicates. Initially, an experienced librarian assisted with the retrieval process. Subsequently, two independent reviewers screened the titles and abstracts for eligible full-text studies. They then examined the full texts according to the inclusion and exclusion criteria to determine their eligibility. Throughout this process, the two independent reviewers (YG and JX worked independently, and in cases of disagreement, a third reviewer (GD) was consulted until consensus was reached. The selection procedure details are illustrated in Figure 1.

### Data extraction and quality assessment

After completing the research selection process, two independent reviewers (YG and JX) collected relevant data from each included study. This data included the study title, participant characteristics, study design, details of the exercise training program, and study outcomes.

The methodology of the included studies was independently evaluated by two reviewers using the Physiotherapy Evidence Database (PEDro) scale. This tool consists of 11 items (Table 2) assessing four methodological domains: randomization, blinding, group comparison, and data analysis. Each item is scored as Yes (1 point) or No (0 points), with the total score excluding qualifying criteria due to their relevance to external validity. The PEDro scale measures methodological quality on a scale from 0 to 10, with higher scores indicating higher quality: 8–10 denotes outstanding quality; 5-7 denotes good quality; 3-4 denotes medium quality; and below 3 denotes poor quality. Additionally, best evidence synthesis (BES) was used to assess the overall body of scientific data. BES classifies evidence into five categories based on methodological quality, the quantity of research, and the consistency of findings (Pluim et al., 2023): Strong evidence: more than two high-quality studies agree on the findings (Brouwers et al., 2015); Moderate evidence: one high-quality study and several low-quality studies agree on the findings (Barbaros et al., 2023); Limited evidence: based on a single study or inconsistent findings from multiple studies (Lambrich and Muehlbauer, 2022); Conflicting evidence: conflicting study findings, with at least 75% consistency among studies (Caballero et al., 2021); No evidence: the finding was not reported in any study.

**TABLE 2:** Results of the methodological quality evaluation of the scale Physiotherapy Evidence Database.

References	Eligibility criteria	Random allocation	Concealed allocation	Groups similar at baseline	Participant blinding	Therapist blinding	Assessor blinding	<15%dropouts	Intention to treat analysis	Between group difference reported	Point estimate and variability reported	PEDro total score
Barber-Westin et al. (2010)	1	0	0	0	0	0	0	1	1	0	1	3
YIDIZ et al. (2019)	1	0	0	0	0	0	0	1	1	0	1	3
Fernandez-Fernandez et al. (2013)	1	1	0	1	0	0	0	1	1	1	1	6
Xiao et al. (2023)	1	1	0	1	0	0	0	1	1	1	1	6
Mainer-Pardos et al. (2024)	1	1	0	1	0	0	0	1	1	1	1	6
Kilit et al. (2019)	1	1	0	1	0	0	0	1	1	1	1	6
Novak et al. (2023)	1	1	0	1	0	0	0	1	1	1	1	6
MoYa-Ramon et al. (2020)	1	1	0	1	0	0	0	0	1	1	1	5
Behringer et al. (2013)	1	1	0	1	0	0	0	1	1	1	1	6
Fernandez-Fernandez et al. (2020)	1	1	0	1	0	0	0	1	0	1	1	5
Shi et al. (2023)	1	1	0	1	0	0	0	1	1	1	1	6
Fernandez-Fernandez et al. (2016)	1	1	0	1	0	0	0	1	1	1	1	6
Fernandez-Fernandez et al. (2024)	1	1	0	1	0	0	0	1	1	1	1	6
Fernandez-Fernandez et al. (2017)	1	1	0	1	0	0	1	1	1	1	1	7
Sinkovic et al. (2023)	1	1	0	1	0	0	0	1	1	1	1	6
Bashir et al. (2019)	1	1	0	1	0	0	0	1	1	1	1	6
Fernandez-Fernandez et al. (2018)	1	1	0	1	0	0	0	1	1	1	1	6
Fernandez-Fernandez et al. (2015)	1	1	0	1	0	0	0	1	1	1	1	6

## Results

### Study selection

A total of 3,400 papers were identified through database searches, as illustrated in Figure 1. The papers were distributed across the following databases: Web of Science (1,292), SportDiscus (1,079), Scopus (803), and PubMed (226). An additional eight studies were identified through Google Scholar and reference lists. After manually removing duplicates, 1795 records remained. These records’ titles and abstracts were reviewed, resulting in 63 papers deemed eligible for full-text analysis. Based on the exclusion criteria, an additional 45 studies were removed. Consequently, 18 RCTs and non-RCTs were included in this review to assess the effect of exercise training on the physical demands of young tennis players.

#### Study quality assessment

The PEDro scores varied from 3 to 7 for the studies included in this review (Table 3). The intra-observer reliability was established at k = 0.81 to 0.83, indicating very high consistency within the same rater across different time points. Similarly, the inter-observer reliability was found to range from k = 0.64 to 0.65, reflecting good agreement between different raters at the same time points. Because of the specificity of tennis training, sixteen publications with a PEDro score of >5, and two with a PEDro score of 3, indicating that the included studies had a high degree of methodological quality and that the research findings were reasonably reliable. Additionally, apart from two studies with a PEDro score of 3, all other studies satisfied the criteria of randomization, baseline similarity of groups, between-group comparison procedures; all of these studies satisfied the point estimate and variability reported; seventeen studies met the criteria for intention to treat analysis; and only one study satisfied the blind assessor criterion, one study dissatisfied <15% dropouts. However, no study has been conducted to justify the use of concealed allocation concealment, blind participants, or blind therapists.

**TABLE 3:** Characteristics of included studies.

Intervention types	Definition criteria	References	Design	Population characteristics	Interventions	Type of exercise training	Measures index	Outcomes
Plyometric training	Plyometric training is a method that utilizes the muscle stretch-shortening cycle, combining rapid and consecutive muscle extensions and contractions to increase muscle strength and enhance power (Moran et al., 2021). e.g., squat jumps	Novak et al. (2023)	EG-CG	n = 30, age = 13.5 ± 1.8years, E.G.,: M: n = 7, F n = 8, CG: M n = 8, F n = 7, TE: NR, DH: NR	Freq.: 2 times/week, time: 30–45min, Length: 6 weeks	Additional plyometric with resistance bands (E.G.,), Control group (CG)	Speed (5m, 10m, 20 m), Power (CMJ, SJ, SLG), Agility (*t*-test, 20 yards)	EG-CG: 5 m→, 10 m→, 20 m→, CMJ↑, SJ→, SLG↑, *t*-test→, 20 yards→
		Behringer et al. (2013)	E.G.,1-CG E.G.,2-CG Pre-post test	n = 33, age = 15.0 ± 1.6years, Sex: M, E.G.,1: n = 10, age = 15.5 ± 0.9years, E.G.,2: n = 13, age = 15.1 ± 1.8years, CG: n = 10, age = 14.6 ± 1.8years, TE: 6.15years, DH: NR	Freq.: 2 times/week, time: 120min, Length: 8 weeks	Plyometric training (E.G.,1), Resistance training (E.G.,2), Control group (CG)	Serve velocity, Serve accuracy, Strength (leg press, chest press, pull down machine, abdominal press)	E.G.,1-CG:Serve velocity↑, Serve accuracy→ E.G.,2-CG: velocity↑, Serve accuracy→ Pre-post test:leg press↑, chest press↑, pull down machine↑, abdominal press↑
		Shi et al. (2023)	EG-CG	n = 16, Sex: M, E.G.,: n = 8, age = 13.7 ± 0.39years, TE: 4.31 ± 0.20years, CG: n = 8, age = 13.6 ± 0.33years, TE: 4.40 ± 0.25years, DH: NR	Freq.: 3 times/week, time: 120min, Length: 12 weeks	Jumping rope training (E.G.,), Control group (CG)	Dynamic balance	EG-CG: Dynamic balance↑
		Fernandez-Fernandez et al. (2016)	Pre-post test	n = 60, age = 12.5 ± 0.3years Sex: M, E.G.,: n = 30, CG: n = 30, TE: NR, DH: RH = 48, LH = 12	Freq.: 2 times/week, time: 30–60min, Length: 8 weeks	Plyometric training (E.G.,), Control group (CG)	Speed (5m, 10m, 20 m), Power (CMJ, SLG, MBT), Agility (505 test), Serve velocity, Serve accuracy	Pre - post: 5 m↑, 10 m↑, 20 m↑, CMJ↑, SLG↑, 505 test↑, MBT↑, Serve velocity↑, Serve accuracy↑
		Sinkovic et al. (2023)	EG-CG	n = 35, age = 12.14 ± 1.3years Sex: M, E.G.,: n = 17, CG: n = 18, TE: NR, DH: NR	Freq.: 2 times/week, time: NR, Length: 6 weeks	Plyometric training (E.G.,), Conditioning training (CG)	Speed (5m, 10m, 20 m), Power (CMJ, SJ, SLG), Agility (*t*-test)	EG-CG: 5 m↑, 10 m↑, 20 m→, CMJ↑, SJ↑, SLG↑, *t*-test→
Neuromuscular training	Neuromuscular training is a comprehensive training method that stimulates peripheral receptors through optimized input signals and cortical integration to enhance communication between the nervous system and muscles (Concha-Cisternas et al., 2023). e.g., T-drill	Barber-Westin et al. (2010)	Pre-post test	n = 15, Sex: M n = 5, F n = 10, age = 13.0 ± 1.5years, TE:>2years, DH:NR	Freq.: 3 times/week, time: 90min, Length: 6 weeks	Neuromuscular training	Speed (baseline forehand, baseline backhand, service line), Agility (Court suicide), Strength (abdominal endurance)	Pre - post: Baseline forehand↑, Baseline backhand↑, service line↑, Court suicide↑, abdominal endurance↑
		Mainer-Pardos et al. (2024)	EG-CG; Pre-post test	n = 12, age = 13.4 ± 0.36years, Sex: M, E.G.,: n = 6, age = 13.6 ± 0.29years, CG: n = 6, age = 13.2 ± 0.31yeaears, TE:>6years, DH: RH = 10, LH = 2	Freq.: 2 times/week, time: 30min, Length: 10 weeks	Neuromuscular training (E.G.,), Control group (CG)	Power (CMJ, DJ, HJ, MBT), Speed (20 m), Agility (180◦COD)	EG-CG: CMJ↑, DJ↑, HJ↑, MBT→, 20 m↑, 180◦COD→ Pre-post test: CMJ↑, DJ↑, HJ↑, MBT↑, 20 m↑, 180◦COD↑
		Fernandez-Fernandez et al. (2018)	Pre-post test	n = 16, age = 12.9 ± 0.4years, Sex: M, E.G.,1: n = 8, E.G.,2: n = 8, TE: 3 ± 1.2years, DH: RH = 13, LH = 3	Freq.: 2 times/week, time: 32.4 ± 7.3min, Length: 5 weeks	Neuromuscular training before tennis specific training (E.G.,1), Neuromuscular training after tennis specific training (E.G.,2)	Speed (5m, 10m, 20 m), Power (CMJ, MBT), Agility (505 test)	Pre - post: E.G.,1:5 m↑, 10 m↑, 20 m↑, CMJ↑, MBT↑, 505↑, E.G.,2: 5 m→, 10 m↑, 20 m→, CMJ→, MBT↑, 505test→
		Fernandez-Fernandez et al. (2020)	EG-CG	n = 29, age = 15.09 ± 1.16years, Sex: M, E.G.,1: n = 14, CG: n = 15, TE: 5.0 ± 1.2years, DH: RH = 27, LH = 2	Freq.: 3 times/week, time: 20–32min, Length: 8 weeks	Neuromuscular warm up (E.G.,), Dynamic warm up (CG)	Speed (5m, 10m, 20 m), Power (CMJ, MBT), Agility (505 test)	EG-CG: 5 m↑, 10 m↑, 20 m→, CMJ↑, MBT↑, 505 test→
		Fernandez-Fernandez et al. (2024)	EG-CG	n = 31, Sex: M, E.G.,: n = 17, age = 16.2 ± 0.4years, CG: n = 14, age = 16.5 ± 0.4years, TE: 7.0 ± 1.2years, DH: NR	Freq.: 2 times/week, time: NR, Length: 6 weeks	Neuromuscular training in sand (E.G.,),Neuromuscular training in hard (CG)	Speed (5m, 10 m), Power (CMJ, RJT), Strength (ABD, ADD), Agility (COD), Dynamic balance	EG-CG: E.G.,: 5 m↑, Dynamic balance↑, ABD↑, ADD↑; CG: CMJ↑, RJT↑, 10 m→, COD→
Functional training	Functional training refers to a method that targets specific parts and connections within the human movement chain to enhance the overall functionality and efficiency of the body (Xiao et al., 2021). e.g., BOSU ball squats	Bashir et al. (2019)	EG-CG	n = 30, age = 15.3 ± 0.8years, Sex: NR, E.G.,: n = 15, age = 15.2 ± 0.41yeaears, CG: n = 15, age = 15.2 ± 0.41yeaears, TE:>1year, DH:NR	Freq.: 3 times/week, time: NR, Length: 8 weeks	Core training (E.G.,), Control group (CG)	Agility (*t*-test), dynamic stability	EG-CG: *t*-test↑, dynamic stability↑
		YILDIZ et al. (2019)	E.G.,1-CG,E.G.,2-CG,E.G.,1-EG2	n = 28, age = 9.6 ± 0.7years, Sex: M, E.G.,1: n = 10, E.G.,2: n = 10, CG: n = 8, TE:3,1 ± 1.3years, DH:NR	Freq.: 3 times/week, time: 45min, Length: 5 weeks	Function training (E.G.,1), Traditional training (E.G.,2), Control group (CG)	Speed (10 m), Agility (*t*-test), Power (CMJ), Flexibility (sit and reach), dynamic stability	E.G.,1-CG:10 m↑, *t*-test↑, CMJ↑, sit and reach↑, dynamic stability↑ E.G.,2-CG: 10 m→, *t*-test→, CMJ↑, sit and reach↑, dynamic stability→ E.G.,1-EG2: 10 m→, *t*-test↑, CMJ↑, sit and reach↑, dynamic stability↑
		Xiao et al. (2023)	EG-CG	n = 40, Sex: M, E.G.,: n = 20, age = 16.7 ± 0.4years, CG: n = 20, age = 16.5 ± 0.6years, TE:>4years, DH:NR	Freq.: 3 times/week, time: 60min, Length: 12 weeks	Function training (E.G.,), Control group (CG)	Strength (push-ups, wall squat), power (over medicine ball throw, standing long jump)	EG-CG: push-ups↑, wall squat↑, over medicine ball throw↑, standing long jump↑
Traditional strength training	Traditional strength training is a method that focuses on increasing muscle mass and strength through repetitive exercises using weights or resistance (González-Badillo et al., 2022). e.g., barbell squat	Fernandez-Fernandez et al. (2013)	EG-CG	n = 30, age = 14.2 ± 0.5years, Sex: M, E.G.,: n = 15, CG: n = 15, TE:>3years, DH: RH = 26, LH = 4	Freq.: 3 times/week, time: 60min, Length: 6 weeks	Strength training (E.G.,), Control group (CG)	Serve velocity, Serve accuracy	EG-CG: Serve velocity↑, Serve accuracy→
HIIT training	HIIT refers to repeated short bouts (less than 45 s) to longer bouts (2–4 min) of high, but not maximal, intensity exercise, or short (10 s or less) repeated-sprint sequences and longer (20–30 s or more) all-out sprints, interspersed with recovery periods (Buchheit and Laursen, 2013). e.g., sprint intervals	Kilit and Arslan (2019)	EG-CG; Pre-post test	n = 29, age = 13.8 ± 0.4years, Sex: M, E.G.,: n = 14, CG: n = 15, TE:>3years, DH: RH = 29	Freq.: 3 times/week, time: 8–16min, Length: 6 weeks	High-intensity interval training (E.G.,) On court tennis training (CG)	Speed (5m, 10m, 20m, 400 m), Power (CMJ, SJ, DJ), Agility (t-drill test)	EG-CG: 5 m→, 10 m→, 20 m→, 400 m↑, CMJ→, SJ→, DJ→, t-drill test↓ Pre-post test:5 m↑, 10 m↑, 20 m↑, 400 m↑, CMJ↑, SJ↑, DJ↑, t-drill test↑
		Moya-Ramon et al. (2020)	EG-CG	n = 20, age = 16.5 ± 0.3years, Sex: M, E.G.,: n = 10, age = 16.7 ± 0.1year, CG: n = 10, age = 16.4 ± 0.3years, TE:9.0 ± 2.6years, DH:NR	Freq.: 2 times/week, time: NR, Length: 6 weeks	Resisted sprint training (E.G.,), Conventional sprint training (CG)	Speed (5m, 10m, 20m, RSA), Power (CMJ, SLJ), Agility (505 test)	EG-CG: 5 m↑, 10 m→, 20 m→, RSA→, CMJ↑, SLJ↑, 505 test→
		Fernandez-Fernandez et al. (2015)	EG-CG; Pre-post test	n = 16, age = 16.9 ± 0.5years, Sex: M, E.G.,: n = 8, CG: n = 8, TE:8.0 ± 2.6years, DH:NR	Freq.: 2 times/week, time: 60 min, Length: 8 weeks	Combined power and repeated sprint training (E.G.,), Control group (CG)	Speed (10m, 20m, 30m, RSA), Power (CMJ)	EG-CG: Pre-post: 10 m↑, 20 m→, 30 m→, RSA↑, CMJ↑
		Fernandez-Fernandez et al. (2017)	EG-CG; Pre-post test	n = 20, age = 14.8 ± 0.1year, Sex: NR, E.G.,: n = 10, CG: n = 10, TE:6.0 ± 1.2years, DH: RH = 16, LH = 4	Freq.: 2 times/week, time: 40 min, Length: 8 weeks	Mixed high-intensity intermittent runs and tennis- specific training (E.G.,), Tennis-specific drills only (CG)	Speed (5m, 10m, 20 m), Power (CMJ), Agility (505 test)	EG-CG: Pre-post: 5 m→, 10 m→, 20 m→, CMJ→, 505 test→

e.g., for example,; Legend: yr, year; M, male, F, female; E.G., experimental group; CG, control group; TE, tennis experience; NR, not reported; DH, dominant hand; RH, right hand; LH, left hand; Freq, frequency; CMJ, countermovement jump; SJ, squat jump; DJ, drop jump; SLG, standing long jump; HJ, jump height; MBT, medicine ball throws; RJT, repeated jump test; ABD, abductor strength; ADD, adductor strength; RSA, repeated sprint ability.; →, no significant changes compared to the control group; ↑, significant changes compared to the control group.

#### Population characteristics

The population characteristics of the eighteen studies included in this review were evaluated on the following aspects (Pluim et al., 2023): sample size: there were 490 participants in the eighteen studies, ranging from 9 to 16 (Brouwers et al., 2015). gender: all eighteen of these studies examined young tennis players. Thirteen research studies focused exclusively on men, two reported on men and women, whereas the remaining three studies did not specify gender (Barbaros et al., 2023); age: none were over the age of 18. The age varied from 9.6 ± 0.7 to 16.9 ± 0.5 (Lambrich and Muehlbauer, 2022). training background: fifteen studies reported the training background of the participants (Caballero et al., 2021); dominant hand: seven studies reported on the dominant hand of participants, while eleven articles did not report on the dominant hand of participants.

### Interventions characteristics

A total of 11 intervention programs are employed in the included studies. These programs encompass neuromuscular training (Fernandez-Fernandez et al., 2024) (Barber-Westin et al., 2010) (Mainer-Pardos et al., 2024) (Fernandez-Fernandez et al., 2018), core training (Bashir et al., 2019), functional training (YILDIZ et al., 2019) (Xiao et al., 2023), strength training (Fernandez-Fernandez et al., 2013), HIIT training (Fernandez-Fernandez et al., 2017) (Kilit and Arslan, 2019), plyometric training (Sinkovic et al., 2023) (Novak et al., 2023) (Behringer et al., 2013) (Fernandez-Fernandez et al., 2016), sprint training (Moya-Ramon et al., 2020), a combination of power and repeated sprint training (Fernandez-Fernandez et al., 2015), neuromuscular warm-up training, dynamic warm-up (Fernandez-Fernandez et al., 2020), rope jumping training (Shi et al., 2023). The duration of the trials covered in the eighteen studies ranged from 6 to 12 weeks. Additionally, the majority of studies use one to three training sessions per week and varied the time of each session from 30 to 90 min.

Based on the titles and abstracts of 16 studies, we categorize the training into five types of interventions: plyometric training, neuromuscular training, functional training, traditional strength training, and HIIT training. Additionally, according to the training plans and rest intervals of two other studies, we include jump rope training under plyometric training and core training under traditional strength training.

## Results by training type

In this systematic review, we assessed the impact of various training modalities on adolescent tennis players across 18 studies. Five studies investigated plyometric training: three enhanced speed, power, and agility; two studies improved strength and serve speed, albeit with varied effects on serve accuracy; and one study enhanced dynamic balance. Five studies utilizing neuromuscular training all reported significant enhancements in speed and agility; four studies examined power and strength, each showing significant improvements; one study reported improvements in dynamic balance. Three studies on functional training demonstrated significant improvements in speed, strength, power, agility, and dynamic balance compared to control groups. Two studies on traditional strength training significantly enhanced strength and power but did not improve speed, agility, or dynamic balance. Additionally, it significantly enhanced serve speed without affecting serve accuracy. Two studies used HIIT in combination with other training, and one study examining HIIT training alone and found significant improvements in speed, power, and agility, but agility is not as good as on court tennis training.

Functional training showed significant improvements in power, agility, and dynamic balance compared to traditional strength training; however, there was no significant difference in speed between the two. Plyometric training significantly improved power and physical demands in 5m and 10 m sprint compared to traditional strength training, but it showed no significant difference in agility, and 20 m speed physical demands was inferior to traditional strength training. Neuromuscular training conducted before conventional training significantly improved speed and power, whereas no improvements were noted when neuromuscular training was performed after conventional training, except for in the 10 m sprint. Training on different surfaces revealed distinct impacts: sand surface training was more effective for speed, dynamic balance and strength, while hard training was superior for power; agility and 10 m sprint showed no difference between the two surfaces. Adding resistance bands to plyometric training significantly improved the power of adolescent tennis players, with no enhancements in speed or agility noted. Incorporating HIIT into conventional tennis training did not yield improvements in speed, power and agility. Additionally, combining power training with repeated sprint training significantly enhanced speed and power compared to repeated sprint training alone. Similarly, resistance sprint training improved power and 5 m sprint physical demands over conventional sprint training, but showed no significant differences for 10m, 20m, repeated sprints, or agility.

## Discussion

This review encompasses 18 studies focused on evaluating the effects of various sports training modalities on the physical demands of adolescent tennis players. The findings demonstrate that plyometric, neuromuscular, and functional training significantly enhance strength, speed, power, agility, and dynamic balance; HIIT significantly increased endurance but did not improve agility; traditional strength training increased power but did not contribute to speed, agility or balance. It is crucial for the training regimen of adolescent tennis players to consider not only the combination and sequence of different training types but also the integration of multiple methods, as these factors critically influence physical demands enhancements.

Compared to conventional tennis training, plyometric, neuromuscular, and functional training significantly enhanced speed, strength, power, agility, and dynamic balance in adolescents. A high level of technical skill is essential in tennis, yet without a robust physical demands base, consistent and sustainable peak physical demands is unattainable (Lambrich and Muehlbauer, 2022). Plyometric training leverages the stretch-shortening cycle to optimize neuromechanical responses, enabling maximal force generation in minimal time by the nervous and muscular systems. This type of training can bridge the gap between strength and speed, improving throwing, jumping and speed physical demands (Eraslan et al., 2020), athletic physical demands in racquet sports (Deng et al., 2023), and children’s physical demands (Marzouki et al., 2022). Neuromuscular training stimulates peripheral receptors to enhance muscle reaction integration and unconscious movement responses via optimized input signals and cortical integration. This training improves dynamic posture control (Concha-Cisternas et al., 2023) and boosts children’s physical demands, particularly in girls (Faigenbaum et al., 2014). Functional training targets specific segments and connections in the human movement chain to execute precise actions. It includes activities like accelerating, decelerating, and stabilizing multi-dimensional motions that align with targeted actions (Xiao et al., 2021). Functional training emphasizes overall human movement training and can adjust joint angles, improve muscle function, and enhance movement efficiency to improve athletic physical demands (Bashir et al., 2022), and has a positive impact on the physical demands of both untrained children and experienced athletes (Bashir et al., 2022) (Li et al., 2023). Compared to conventional tennis training, HIIT better improves endurance but has poorer agility (Kilit and Arslan, 2019), traditional strength training increased power but did not contribute to speed, agility or balance. HIIT enhances the cardiovascular and metabolic systems through short bursts of high-intensity exercise, improving cardiorespiratory function and both aerobic and anaerobic muscle metabolism (Foster et al., 2015). These physiological adaptations significantly boost endurance performance. However, agility training requires rapid neuromuscular responses and coordination (Chaouachi et al., 2012). Traditional strength training induces neuromuscular adaptations such as improved intra- and inter-muscular coordination, increased muscle-tendon stiffness, and enhanced motor unit recruitment and firing rates, along with morphological changes (Llanos-Lagos et al., 2024). It effectively enhances adolescent power and indirectly improves sprinting and jumping abilities, but does not improve speed, agility, or balance (Westblad et al., 2021). In conclusion, plyometric, neuromuscular, and functional training significantly enhance speed, strength, power, agility, and dynamic balance in adolescents more than traditional tennis training, HIIT is more effective in developing endurance compared to traditional tennis training, and strength training excels in enhancing power over conventional tennis drills.

In addition to the variance in the effects of different training types compared to traditional tennis training on adolescent physical demands, discrepancies also exist among the different training modalities themselves. Functional training, which emphasizes movement patterns, stability, and power rather than isolating specific muscle groups like traditional strength training, significantly enhances power, agility, and balance, but not speed (Yildiz et al., 2019). This findings diverge from previous research, where 8 weeks of functional training improved speed and power more markedly in dragon boat athletes compared to traditional strength training, although it did not significantly enhance strength or agility (Wu et al., 2023). Despite there are differences in sports events, this discrepancy underscores the need for more comprehensive evidence to ascertain the differential effects of functional and traditional strength training in adolescent tennis players. Plyometric training has proven more effective than traditional strength training in enhancing adolescent power and speed (Sinkovic et al., 2023). In boys aged 11–16, maximal speed is determined by the ability to generate high levels of force within a very short ground contact time (approximately 140 milliseconds), while resisting vertical displacement of the center of mass and propelling the body forward (Meyers et al., 2019). Plyometric training, known for influencing stretch-shortening cycle activities, provides optimal responses for rapid, explosive actions, significantly enhances power and speed in adolescents more effectively than traditional strength training (Posnakidis et al., 2022) (Behm et al., 2017). Therefore, improving agility and balance is more suited to the use of functional training and improving power and speed is more suited to the use of plyometric training than strength training.

Different training sequences and surfaces can significantly impact the development of physical demands in adolescent tennis players. The timing of neuromuscular training can greatly affect its efficacy. Conducting neuromuscular training before regular tennis practice, rather than after, more effectively enhances speed, agility, and power (Fernandez-Fernandez et al., 2018). This enhancement is likely due to optimal neural and muscular preparation provided by pre-training sessions, facilitating better muscle fiber activation and recruitment during subsequent exercises. In contrast, neuromuscular training performed after routine practices may induce fatigue, reducing potential benefits in speed, agility, and power (Hammami et al., 2018). The choice of training surface also significantly impacts an athlete’s physical demands. Training on hard surfaces is better suited for improving explosive power (Fernandez-Fernandez et al., 2024) as it provides stable support and minimizes energy loss (Peitz et al., 2018). Conversely, sand training, with its inherent instability, increases muscle loading (Impellizzeri et al., 2008), making it more effective for enhancing strength, speed, and balance demands (Fernandez-Fernandez et al., 2024). However, both surfaces are equally effective in improving agility with no significant differences observed. Optimally utilizing these training environments and methods can significantly enhance training outcomes. Therefore, for optimal enhancement of speed, flexibility, and strength, it is advisable to conduct neuromuscular training before routine sessions; choose hard surfaces for improving explosive power, and sand surfaces for better strength, speed, and balance.

The effectiveness of combining different training methods to enhance the physical demands of adolescent tennis players varies. Incorporating resistance bands in plyometric training significantly enhances power but has minimal impact on speed and agility (Novak et al., 2023). The additional resistance can increase power but may also prolong ground contact time during rapid stretch-shortening cycle movements, which can affect improvements in speed and flexibility (Duchateau and Amiridis, 2023). HIIT combined with power training significantly outperforms traditional HIIT in 10-m sprints and power, with no notable differences in 20-m and 30-m sprints (Fernandez-Fernandez et al., 2015). HIIT integrated with strength training improves speed in 5-m sprints and power over traditional HIIT, but it does not enhance 10-m and 20-m sprints speed, repeated sprints speed and agility (Moya-Ramon et al., 2020). The benefits of combining HIIT with power and strength training likely arise from enhanced muscular strength and neuromuscular adaptability, directly improving physical demands related to explosive power and strength (Chang et al., 2022). Traditional HIIT, focused primarily on aerobic endurance, tends to overshadow the benefits of strength training in tests where endurance is increasingly demanded. Compared to exclusive tennis-specific training, combining specialized tennis training with high-intensity interval running does not yield improvements in speed, power, or agility (Fernandez-Fernandez et al., 2017). The cardiorespiratory endurance benefits of high-intensity interval running may require endurance physical demands metrics for detection. Therefore, for specific training goals and athletic needs, particularly when aiming to enhance short-distance sprinting, repeated sprint ability, and power, integrating HIIT with power and strength training is the superior choice.

This study also has several limitations. First, it only integrates studies comparing functional and plyometric training with traditional strength training and does not comprehensively clarify the advantages and disadvantages between various training modalities. Due to a lack of sufficient research in this area and differences in study designs, it is not possible to conduct a network meta-analysis to obtain higher-level evidence for determining the optimal training approach. Second, due to the limited number of relevant studies, we have not discussed in detail the effects of different age groups, training years or training durations on training outcomes. It would be very valuable if future research could determine which training methods are most effective for specific age groups or levels of experience. Third, as a systematic review of existing studies, this research does not provide definitive conclusions on how to effectively integrate different types of training. Additionally, our review was limited to studies published in English. The exclusion of unpublished data, gray literature, and relevant research in other languages might have introduced bias, potentially leading to an incomplete or skewed representation of the true effects of the training methods evaluated. Lastly, while this review considers the differences between various training types and offers recommendations based on training objectives, it does not provide specific details or guidelines on the content of each training method. The differences that specific training details may produce also warrant further investigation.

## Conclusion

In conclusion, the factors influencing the effectiveness of physical training for adolescent tennis players are multifaceted. When selecting training methods to enhance the physical demands of speed, strength, power, agility, and dynamic balance in adolescents, plyometric training, neuromuscular training and functional training should be prioritized over traditional tennis training, while HIIT is preferable for enhancing endurance. Functional training is particularly suitable for improving flexibility and balance, while plyometric training is more effective for enhancing power and speed. Neuromuscular training performed before routine workouts is the better choice for improving speed, flexibility, and strength. Hard surface training should be used to increase explosiveness, whereas sand training is more appropriate for enhancing strength, speed, and balance. Additionally, combining HIIT with strength training is particularly beneficial when there is a need to improve short-distance sprinting, repeated sprint ability, and power, according to specific training goals and athletic requirements. Appropriately combining and utilizing these training methods can comprehensively and optimally enhance the overall physical demands and sports performance of adolescent tennis players.

## Data Availability

The original contributions presented in the study are included in the article/supplementary material, further inquiries can be directed to the corresponding authors.
